# Unmasking Intra-Tumoral Heterogeneity and Clonal Evolution in NF1-MPNST

**DOI:** 10.3390/genes11050499

**Published:** 2020-05-01

**Authors:** Chang-In Moon, William Tompkins, Yuxi Wang, Abigail Godec, Xiaochun Zhang, Patrik Pipkorn, Christopher A. Miller, Carina Dehner, Sonika Dahiya, Angela C. Hirbe

**Affiliations:** 1 Division of Medical Oncology, Department of Medicine, Washington University School of Medicine, St. Louis, MO 63110, USA; moonchangin@wustl.edu (C.-I.M.); yuxi.w@wustl.edu (Y.W.); zhang.x@wustl.edu (X.Z.); 2 Washington University School of Medicine, St. Louis, MO 63110, USA; wtompkins@wustl.edu; 3 College of Human Medicine, Michigan State University, East Lansing, MI 48824, USA; godecabi@msu.edu; 4 Department of Otolaryngology, Division of Head and Neck Surgery, Washington University School of Medicine, St. Louis, MO 63110, USA; ppipkorn@wustl.edu; 5 Siteman Cancer Center, St. Louis, MO 63110, USA; c.a.miller@wustl.edu (C.A.M.); sdahiya@wustl.edu (S.D.); 6 McDonnell Genome Institute, Division of Oncology—Stem Cell Biology, Department of Medicine, Washington University School of Medicine, St. Louis, MO 63110, USA; 7 Department of Pathology and Immunology, Washington University School of Medicine, St. Louis, MO 63110, USA; cdehner@wustl.edu

**Keywords:** NF1, MPNST, genomics, heterogeneity

## Abstract

Sarcomas are highly aggressive cancers that have a high propensity for metastasis, fail to respond to conventional therapies, and carry a poor 5-year survival rate. This is particularly true for patients with neurofibromatosis type 1 (NF1), in which 8%–13% of affected individuals will develop a malignant peripheral nerve sheath tumor (MPNST). Despite continued research, no effective therapies have emerged from recent clinical trials based on preclinical work. One explanation for these failures could be the lack of attention to intra-tumoral heterogeneity. Prior studies have relied on a single sample from these tumors, which may not be representative of all subclones present within the tumor. In the current study, samples were taken from three distinct areas within a single tumor from a patient with an NF1-MPNST. Whole exome sequencing, RNA sequencing, and copy number analysis were performed on each sample. A blood sample was obtained as a germline DNA control. Distinct mutational signatures were identified in different areas of the tumor as well as significant differences in gene expression among the spatially distinct areas, leading to an understanding of the clonal evolution within this patient. These data suggest that multi-regional sampling may be important for driver gene identification and biomarker development in the future.

## 1. Introduction

Malignant peripheral nerve sheath tumor (MPNSTs) is the sixth most common soft tissue sarcoma [1] and has an incidence rate of 0.1–0.2 per 100,000 persons per year [2]. MPNSTs are often associated with neurofibromatosis type 1 (NF1). The incidence rate of MPNSTs in patients with NF1 is much higher than that of the general population, estimated to be 1.6 per 1000 per year, or a lifetime risk of 8–13% [3]. Approximately 50% of MPNSTs occur in patients with neurofibromatosis [4,5,6,7], and the other 50% of MPNSTs occur sporadically or in the setting of previous radiation therapy [4,6]. In the setting of NF1, MPNSTs often arise within a pre-existing benign nerve sheath tumor (plexiform neurofibroma) [4,7]. 

Prognosis remains poor for patients with MPNST despite multi-modality therapy [2,5,6,7,8,9,10]. In the setting of metastatic disease, treatment is limited to cytotoxic chemotherapy, typically consisting of single agent doxorubicin or a combination of doxorubicin and ifosfamide [11,12,13]. 

A number of different genes have been implicated in the development of MPNSTs. One of the most commonly used models for preclinical testing was developed by Cichowski et al. and Vogel et al; they demonstrated that mice with germline variants in *Nf1* and *Tp53* develop MPNSTs, supporting a cooperative and causal role for these tumor suppressors in the context of MPNST formation [14,15]. Other groups have found a reduction in expression of *PTEN*, a tumor suppressor in the *PI3K/AKT/mTOR* pathway, in MPNSTs compared to benign nerve sheath tumors in a manner that is not regulated by *NF1* [16]. Keng et al. went on to demonstrate the cooperative roles of *Pten* and *Nf1* in the tumorigenesis of MPNSTs in vivo with transgenic mouse models [17]. Gregorian et al. further elucidated the cooperative relationship between *k-ras* activation and *Pten* deletion, showing that both variants in combination led to 100% penetrable development of MPNSTs [18]. Another gene implicated in MPNST pathogenesis is *INK4A*, a tumor suppressor encoding both *p16* and *p19*. Deletions in this gene have been identified in MPNSTs but not in benign neurofibromas [19]. Lu et al. demonstrated a difference in aberrant expression of *ATRX*, a DNA helicase that plays a role in chromatin regulation and maintenance of telomeres, between MPNSTs and benign neurofibromas [20]. Additionally, variants in *EED* and *SUZ12* have been observed in MPNST. These genes code for components of the PRC2 complex which is involved in transcriptional repression. Lee et al. showed loss-of-function somatic alterations of PRC2 components in 92% of sporadic, 70% of NF1-associated and 90% of radiotherapy-associated MPNSTs. Further, introduction of the lost PRC2 component in a PRC2-deficient MPNST cell line decreased cell growth [21]. Others have found alterations such as structural alterations of *PDGFRA* (platelet-derived growth factor-α) in 26% of MPNST samples [22]; increased expression of *EGF-R* (epidermal growth factor receptor) by immunohistochemistry in MPNSTs [23]; and *IGFR1* gene amplification in 24% of MPNSTs [24]. 

Despite all of this research, no effective therapies have emerged from recent clinical studies based on this genomic data and subsequent preclinical studies. Intra-tumoral heterogeneity is a possible reason for these shortcomings. Prior studies have relied on a single sample from these tumors. All the subclones within a tumor may not be captured by this approach. Our aim in this study is to investigate intra-tumoral heterogeneity more thoroughly through analysis of samples taken from multiple sites of the same MPNST.

## 2. Materials and Methods

### 2.1. Study Approvals

Blood and tumor were obtained from an individual diagnosed with NF1 according to established criteria [25] and treated for a MPNST at Washington University/St. Louis Children’s Hospital NF Clinical Program (St. Louis, MO, USA). The human tumor samples were collected under an approved IRB protocol (#201203042) at Washington University, and the patient was appropriately consented.

### 2.2. Sample Collection

**Figure 1:**
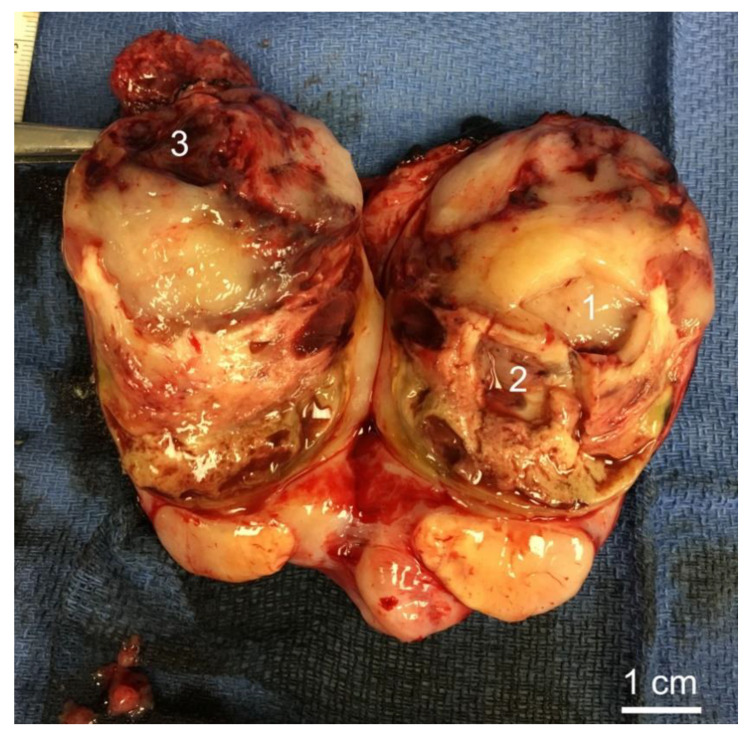
Malignant peripheral nerve sheath tumor (MPNST) sampled areas. Area 1 shows an area centrally located in MPNST, Area 2 an area of hemorrhage, and Area 3 an area of necrosis.

Samples were taken from three distinct areas within a single tumor from a patient with an NF1-MPNST immediately after surgical resection with guidance from a pathologist (SD). While area “1” represented solid, tan homogeneous tumor lacking hemorrhage and/or necrosis, areas “2” and “3” of the tumor grossly appeared necrotic and hemorrhagic respectively. 20 g of tissue was taken from each area. Each area was then divided to be used for RNA extraction, DNA extraction, and slide preparation to analyze the histology. A gross image of the tumor was taken at this time and is shown as Figure 1. 

### 2.3. Histology

Images of the hematoxylin-eosin sections were taken (20X magnification) using an Olympus BX-51 microscope using an Olympus DP71 digital camera, and DP Controller software. Tumor purity was estimated based on morphologic review of the entire hematoxylin-eosin stained section estimating the number of tumor cells, stromal cells, lymphocytes, and extravasated red blood cells. Two pathologists reviewed these slides independently providing an estimated percentage of total tumors cells per slide.

### 2.4. Sequencing and Bioinformatics Analysis

Whole exome sequencing (WES), RNA sequencing (RNA-Seq), and copy number analysis (CNVkit) [26] were performed on each sample and compared to a blood sample as a germline DNA control. Both Illumina Whole Genome Sequencing (eWGS) of 3 tumor samples and 1 PBMC normal sample, and Illumina RNA Sequencing of the 3 tumor samples were generated from the sampled areas.

#### 2.4.1. Library Construction and Sequencing

Each tumor had 2 enriched libraries constructed (n = 6), and the PBMCs had a single enriched library constructed (n = 1). Exome libraries were captured with an IDT exome reagent, then pooled with a WGS library for sequencing on an Illumina HiSeq4000 with at least 1000x coverage. RNA was prepared with a TrueSeq stranded total RNA library kit, then sequenced on an Illumina HISeq4000 with 72M reads per sample.

#### 2.4.2. IDT Exome Sequencing Variant Detection

Genomic data were aligned against reference sequence hg38 via BWA-MEM [27] with Base Quality Score Recalibration (BQSR). Structural variants (SVs) and large indels were detected using manta [28]. SNVs and small indels were detected using VarScan2 [29], Strelka2 [30], MuTect2 [31], and Pindel [32] via the somatic pipelines available at https://github.com/genome/analysis-workflows, which includes best-practice variant filtering and annotation with VEP (Variant Effect Predictor, version 95) [33]. Manual review was used to remove additional sequencing artifacts. Germline variants and somatic variants reported on variant detecting pipeline were compared to see any intersection of variants. Any intersecting variants were removed from the somatic variant gene list, thus filtering out the germline variants. Common variants with 1000 genome MAF (minor allele frequency) > 0.05 were filtered out. Waterfall somatic variant plots were created with GenVisR [34] by including somatic variants that occurred in each area. Variants reported on the waterfall plot are most likely to be pathogenic, which is reported via VEP. These variants were not reported as a somatic variant in COSMIC (Catalogue Of Somatic Mutations In Cancer) [35] and ClinVar [36] archive, thus these variants are best classified as variants with unknown significance. In order to predict clinical significance and predictions of the functional effects of these variants, each variant was reviewed on SIFT [37] and Polyphen [38]. IMPACT rating was determined by VEP for each non-coding variant.

#### 2.4.3. Copy Number Analysis

CNVkit was used to infer and visualize copy number from high-throughput DNA sequencing data. Coverage for each bait position in the exome reagent was calculated, then segments of constant copy number were identified using circular binary segmentation. Data were plotted to provide visualization of CNVs.

#### 2.4.4. Inference of Clonal Phylogeny

SciClone [39] and ClonEvol [40] were utilized to attempt to perform a phylogeny inference. However, the analysis was complicated by the abundance of copy number-altered regions in these tumors, and these standard algorithms were unable to automatically perform that inference. Manual review of the shared and private single nucleotide variants and large copy number altered areas, though, revealed only one possible phylogeny for this tumor.

#### 2.4.5. RNA Sequence Preprocessing

RNA-Sequence (RNA-seq) was trimmed from 3′-end with a minimum quality Phred score of 20 and aligned against hg38—Ensembl Transcripts release 99 via BWA-MEM. Pre/post quality control and full expectation-maximization (EM) quantification were run via Partek^®^ Flow^®^ [41]. Gene counts and transcript counts were normalized by CPM (counts per million) by using edgeR [42] package. Heatmap visualizations were created using gplots [43] R package (Warnes, G.R. Seattle, WA, USA).

#### 2.4.6. Gene Differential Expression Analysis

The gene-specific analysis (GSA) method was used to test for differential expression of genes or transcript between sample regions in Partek^®^ Flow^®^ [44]. Differential expressed genes were defined as the following statistic parameters: *p*-value <= 0.05; FDR step up <= 0.05; Fold Change < −2 or >2. From differentially expressed genes, a GO enrichment test was used to functionally profile this set of genes, to determine which GO terms appear more frequently than would be expected by chance when examining the set of terms annotated to the input genes, each associated with a *p*-value.

#### 2.4.7. Pathway Analysis

A list of genes in copy number aberrant (CNA) regions was extracted. CNA regions were defined as copy number regions greater than 3 or copy number regions less than 1. For each area, we intersected the list of genes that are located in the CNA regions with the differentially expressed gene list reported in the RNA differential expression analysis (*p*-value <= 0.05). PantherDB [45] was utilized to discover GO terms and pathways that may be affected by these genes.

## 3. Results

### 3.1. Patient Information

Patient characteristics can be seen in Table 1. The patient was a male with a history significant for a clinical diagnosis of neurofibromatosis type 1—patient had a plexiform neurofibroma, spinal neurofibromas, café au lait macules, and multiple first-degree relatives with neurofibromatosis type 1—and was 40 years old at the time of diagnosis of MPNST. He presented with a large tumor located in the left neck. Resection showed a high-grade malignant peripheral nerve sheath tumor, 10.2 cm in the largest dimension, with negative margins. The patient did not receive any adjuvant therapy for his MPNST following initial resection due to poor performance status. He recurred 21 months after the initial diagnosis and ultimately died secondary to complications from metastatic disease (33 months after initial diagnosis). Samples were taken in three different locations within the primary tumor immediately following the inititial resection for the purpose of this study. 

### 3.2. Histology of Biopsy Sites

**Figure 2:**
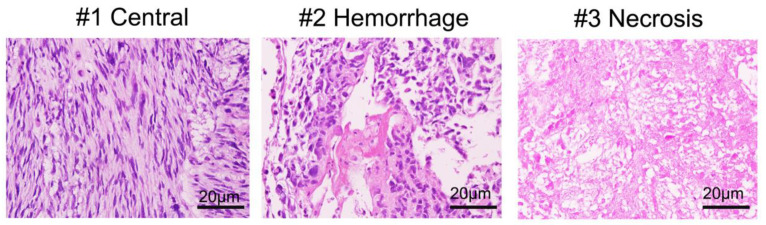
H&E stained sections of the biopsy sites. H&E stained sections (20X) show areas (#1) of relatively uniform, spindled cells with fascicular growth pattern, characteristic for MPNST. Sampled area #2 shows evidence of hemorrhage within the tumor, a feature commonly seen in MPNST. Area #3 shows abundant tumor necrosis.

We first reviewed the H&E images of the tumor to correlate histology to the gross images of the tumor. H&E stained sections in Figure 2 show representative images of the three sampled areas. Area #1 demonstrates tissue of a spindle cell neoplasm of neural differentiation arranged in fascicles with elongated hyperchromatic nuclei and a mild to moderate amount of cytoplasm. The tumor purity of this sample was >95%. Area #2 shows spindled cells in a background of hemorrhage, a finding commonly seen in these high-grade tumors with a tumor purity of >95%. Area #3 represents an area of necrosis, another characteristic finding for MPNST. This sample showed >95% tumor purity.

### 3.3. Whole Exome Sequencing (WES), RNA Sequencing (RNA-Seq), and Copy Number Analysis

**Figure 3:**
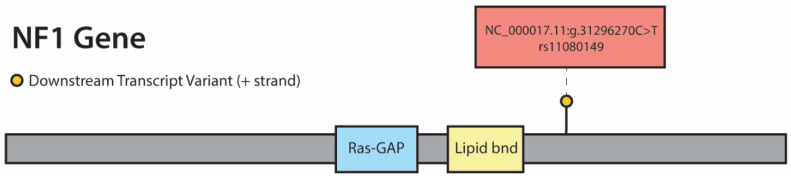
Location of NF1 germline variant. One intronic germline variant, NC_000017.11:g.31296270C>T (rs11080149). was identified and is depicted in this figure.

**Figure 4:**
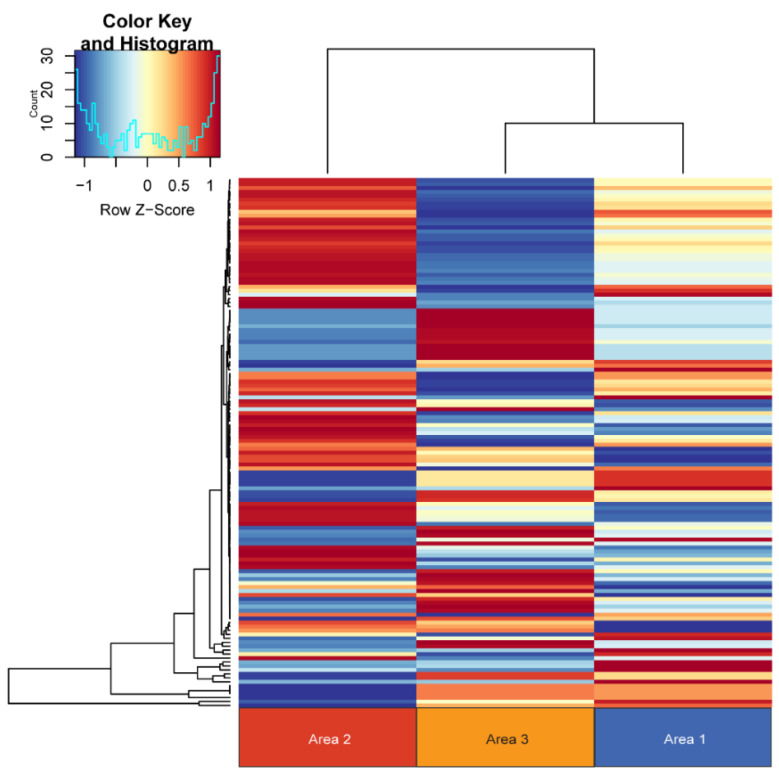
RNA-Seq Heatmap. Normalized read counts by counts per million (CPM) in differentially expressed genes are depicted here. Distinct gene expression profiles can be appreciated in each biopsied area. Each column is depicted as list of genes.

We first interrogated the sequencing data to identify the germline NF1 variant within this tumor. Figure 3 shows a lollipop plot identifying the patient’s likely NF1 germline variant based on exclusion of any variants with minor allele frequency >0.05 in the 1000 genomes database. Next, to investigate intra-tumoral heterogeneity within the sample, RNA sequencing of the three sample sites was performed and is shown in Figure 4. 

**Figure 5:**
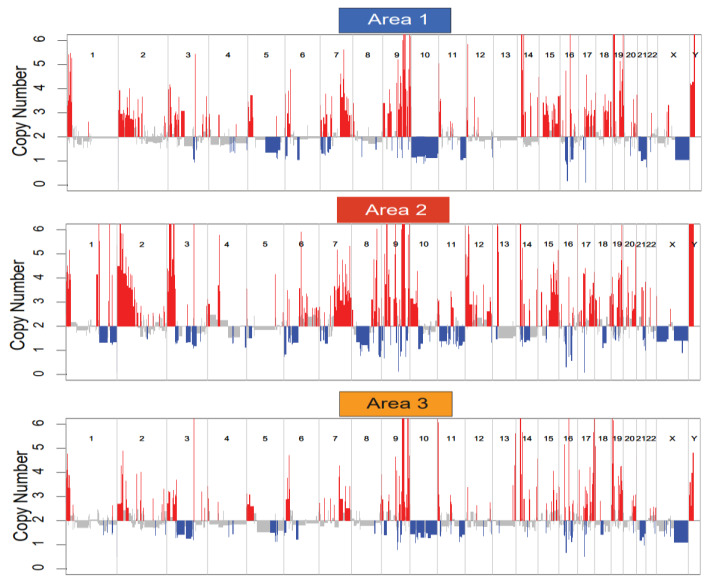
Copy Number Variation Plot. Copy number variation plots for each biopsied site demonstrate distinct copy number signatures.

**Figure 6:**
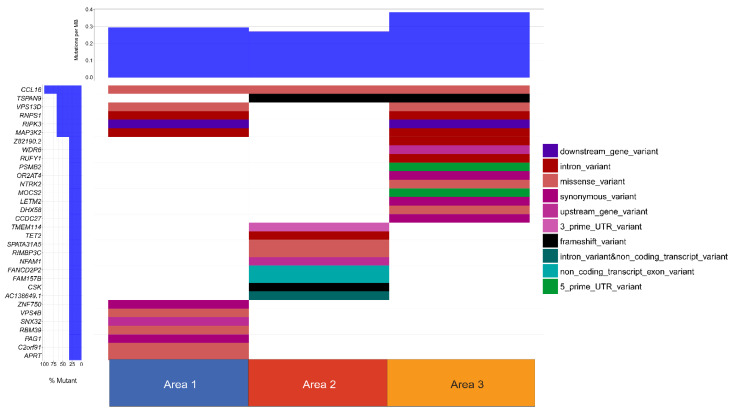
Somatic Variant Waterfall Plot. All somatic variants displayed on a waterfall plot. Each row represents a gene. Distinct somatic variant signatures are appreciated.

**Figure 7:**
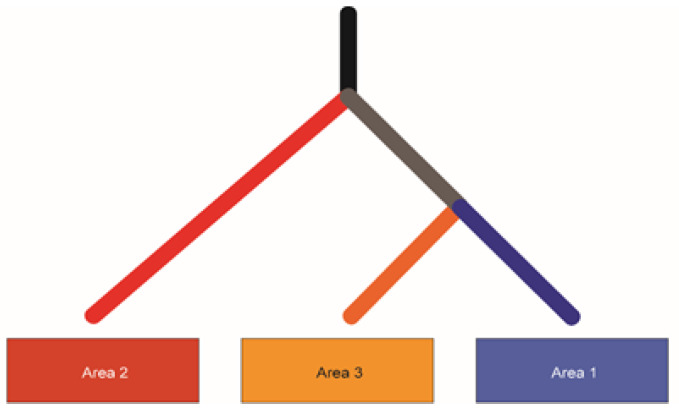
Phylogenetic Tree. A predicted phylogenetic tree of the tumor subclones.

Distinct gene expression profiles were observed in each of the areas sampled. The top 16 differentially expressed genes are listed in Table 2 and include a number of genes involved in transcription and translation. We next performed a copy number analysis of the three biopsy sites to determine whether or not different copy number alterations were observed in each area (Figure 5). Distinct copy number signatures can be appreciated in each of the three samples further illustrating intra-tumoral heterogeneity. Additionally, we evaluated the single nucleotide variants found in each of the samples. This broad overview of all somatic variants is depicted in the waterfall plot in Figure 6. Again, distinct somatic variants can be appreciated across different areas. We next explored the potential significance of these variants through further bioinformatics analysis. While the biological significance of each of these variants is uncertain, there is evidence that some of these variants may play a role in the pathogenesis. For each variant in a coding region, CBioPortal [47] was queried for each gene to determine if the somatic variant was in a functional domain. Additionally, the RNAseq data was queried to determine if the variant in a specific area of the tumor influenced the gene expression of that gene in a specific area. Finally, SIFT and Polyphen were used to predict pathogenicity. Table 3a,b list the somatic variants in the coding region that may play a role in the pathogenesis of this tumor based on the above criteria. For those mutaions in non-coding regions, the Ensembl Variant Effect Predictor [33] was used to determine whether or not the variant would be predicted to affect gene expression. All of the identified variants were classified as modifiers, indicating that pathogenicity prediction is difficult, thus the effects of these variants are unclear. (Table 3c). Further details of the somatic variants can be found in Supplemental Table S1. Next, a gene ontology analysis was performed. To do this, a list of genes in copy number aberrant (CNA) regions was extracted. For each area, the list of genes located in the CNA regions intersected with the differentially expressed gene list reported in the RNA differential expression analysis, and PantherDB [45] was utilized to identify pathways that may be affected by these genes. Table 4 displays the unique genes in each area with copy number aberrations and alterations in gene expression. Genes depicted in Area 1 have been reported in the literature to serve a myriad of functions in tumorigenesis, including base excision repair, nucleotide excision repair, and alternative splicing [48,49,50,51,52,53,54,55]. Those in Area 2 are involved in several different pathways, including transcriptional regulation in addition to ribosomal and proteasomal function [56,57,58,59,60]. Finally, the genes in Area 3 consist of several ribosomal subunits and small nucleolar RNAs, suggesting that both translation and transcription are uniquely affected compared to other areas [61,62,63]. This analysis suggests that there may be different functional programs at play across the three areas. Next, we manually reviewed the data to look for changes in other known drivers of MPNST including TP53, ATRX, EED, SUZ12, and CDKN2A. There were no copy number changes or somatic mutions in any of these genes. Finally, we performed a careful manual review of all of the shared and unique somatic variants and copy number alterations in each area in order to develop a predicted clonal evolution. Figure 7 depicts the predicted phylogenetic tree of the subclones from each area, representing the likely clonal evolution of the tumor. 

## 4. Discussion

Despite advances in our understanding of the pathobiology of MPNST and the identification of seemingly promising therapeutic targets using a single model system in preclinical studies, no investigational agents have demonstrated efficacy following translation to human clinical trials. One element that has largely been ignored in the study of MPNST has been the possible existence of intra-tumoral heterogeneity. No single study in MPNST has focused on intra-tumoral heterogeneity. However, spatial intra-tumoral heterogeneity has become an area of interest in the study of other solid malignancies to begin to understand clonal evolution [91,92,93,94,95]. Within the NF1 field, researchers are beginning to appreciate the importance of understanding spatial and temporal heterogeneity. For example, Peacock et al. performed a genomic analysis of serial samples from one patient who developed an MPNST. Samples were taken at four timepoints (benign plexiform neurofibroma, MPNST pre-treatment, MPNST post-treatment, and MPNST at time of metastasis) [96]. They observed early hemizygous microdeletions in *NF1* and *TP53* with progressive amplifications of *MET*, *HGF*, and *EGFR,* highlighting the potential role of these pathways in progression. Additionally, Carriό et al. have started to examine intra-tumoral heterogeneity in PNF (plexiform neurofibromas), ANF (atypical neurofibroma) and ANNUBP (atypical neurofibromatous neoplasms with uncertain biological potential), the precursors to MPNST. They performed SNP-array analysis and exome sequencing on multiple biopsies of eight PNF, of which some had areas consistent with ANF or ANNUBP. Their data suggested that loss of a single copy of *CDKN2A/B* in *NF1* null cells is sufficient to start ANF development and that total inactivation of both copies is necessary to form ANNUBP [97]. Our study represents the first look at spatial intra-tumoral heterogeneity within an MPNST. We have demonstrated differing mutational profiles, copy number alteration signatures, and gene expression profiles within the three areas sampled. The differing mutation profile includes a variety of single nucleotide variants, including missense, frameshift, and synonymous variants. The role of synonymous variants in the tumorigenesis of MPNST is uncertain. However, there is increasing evidence that synonymous variants can alter gene expression and protein function and thus cannot be simply disregarded [98,99,100,101]. Additionally, several of the genes in Table 3a,b have previously been implicated in cancer [102,103,104,105,106,107,108,109,110,111,112,113,114,115]. For example, in Area 2, *CSK* was found to have a frameshift variant in its functional domain. *CSK* encodes a C-terminal Src kinase that has previously been found to act as a tumor suppressor in both breast cancer and prostate cancer [112,113,114]. Interestingly, in the context of breast cancer, Smith et al. showed that C-terminal Src kinase loss facilitated tumorigenesis by altering expression of the *PRC2* complex subunits, *EZH2* and *SUZ12* [113]. Based on these data, it is possible that alterations in *CSK* could be another way in which the *PRC2* complex is affected in MPNST. Another gene, *CCL16*, is involved in chemotaxis of human monocytes and lymphocytes. This chemokine was shown to delay mammary tumor growth and reduce rates of metastasis in mouse models [115], raising the possibility of decreased immune surveillance of our patient’s MPNST secondary to a non-functional *CCL16*. In addition to the differences in single nucleotide variants, there were differences in copy number alterations across the three areas with Area 2 showing the most distinct signature in terms of copy number gains and losses. The degree to which each somatic variant, differentially expressed gene, and copy number aberration contributes to the biologic heterogeneity of the tumor remains uncertain. However, future work in our lab will be geared at elucidating this information. Finally, there was a distinct difference in gene expression among the three areas with gene ontology studies pointing toward differences in translation and protein targeting. 

Taken together, these data point toward the existence of intra-tumoral heterogeneity and suggest that further investigation into this phenomenon is warranted. Additionally, these data suggest that there should be some caution taken in interpreting sequencing that comes from a single biopsy site. The advent of single cell sequencing has allowed for more rigorous evaluation of intra-tumoral heterogeneity in other cancers including acute leukemias [116,117], as well as in some solid malignancies [118,119]. Future work will be geared at using this data as the foundation to better understand clonal heterogeneity along with single cell sequencing to comprehensively evaluate intra-tumoral heterogeneity and clonal evolution of MPNST.

## 5. Conclusions

Significant intra-tumoral heterogeneity exists and may be a barrier to our ability to improve outcomes in patients with NF1-MPNST. These data suggest that multi-regional sampling may be necessary to understand clonal evolution, and for driver gene identification and biomarker development in the future.

## Figures and Tables

**Table 1:** Patient Characteristics.

Age at Diagnosis, Years	Sex	Tumor Location	Tumor Size/Grade	Surgical Margin Status	Disease Status	Metastasis	Adjuvant Treatment	OS *, Months
40	Male	Left neck	10.2 cm, Grade 3 ^1^	Negative	Recurred	Lung	None	33

^1^ By French Federation of Cancer Centers Sarcoma Group Grading System (FNCLCC) [46]; * OS = Overall Survival-time from diagnosis of MPNST to death.

**Table 2:** Top Differentially Expressed Genes. The gene-specific analysis was used to test for differential expression of genes or transcript between sample regions in Partek^®^ Flow^®^. Statistical cutoff are made by these following parameters: *p*-value <= 0.05; FDR step up <= 0.05; Fold Change <−2 or >2.

Gene Symbol	*p*-Value (1 vs. 2)	Fold Change (1 vs. 2)	*p*-Value (1 vs. 3)	Fold Change (1 vs. 3)	*p*-Value (2 vs. 3)	Fold Change (2 vs. 3)
*EEF1A1*	2.04 × 10^−84^	−3.32	3.33 × 10^−16^	2.20	1.35 × 10^−119^	7.31
*RPS27*	4.32 × 10^−24^	−2.51	7.64 × 10^−13^	3.01	4.27 × 10^−46^	7.55
*RPS27A*	1.69 × 10^−12^	−2.62	9.42 × 10^−05^	2.27	4.16 × 10^−21^	5.95
*H3C3*	7.46 × 10^−12^	−4.51	5.05 × 10^−04^	11.2	5.54 × 10^−09^	50.6
*RPLP1*	2.36 × 10^−10^	−2.57	7.25 × 10^−04^	2.13	2.43 × 10^−17^	5.48
*SNORD13*	3.24 × 10^−10^	3.00	8.25 × 10^−62^	−4.91	3.52 × 10^−66^	−14.8
*RPLP0*	1.05 × 10^−09^	−2.26	1.60 × 10^−04^	2.09	1.73 × 10^−18^	4.72
*TPI1*	1.65 × 10^−08^	−2.27	5.61 × 10^−04^	2.08	6.52 × 10^−16^	4.72
*RPL23AP42*	3.77 × 10^−07^	−2.21	8.40 × 10^−04^	2.16	8.65 × 10^−14^	4.78
*RPS23*	5.34 × 10^−06^	−2.46	1.17 × 10^−03^	2.92	9.16 × 10^−11^	7.19
*MT-TI*	4.64 × 10^−05^	3.44	1.19 × 10^−15^	−3.67	6.36 × 10^−20^	−12.6
*SNORA81*	2.28 × 10^−04^	33.3	4.00 × 10^−11^	−3.39	5.12 × 10^−07^	−11.3
*RNY1*	2.45 × 10^−04^	2.65	4.67 × 10^−24^	−4.71	8.10 × 10^−27^	−12.5
*RNVU1-31*	5.00 × 10^−04^	−4.18	3.83 × 10^−14^	−17.7	7.07 × 10^−13^	−4.23
*MT-TM*	6.37 × 10^−04^	3.70	2.29 × 10^−07^	−2.89	2.69 × 10^−11^	−10.7
*TMSB4XP6*	1.16 × 10^−03^	3.19	2.89 × 10^−04^	−2.15	7.91 × 10^−09^	−6.87

**Table:** 

(a)							
Gene	Area	Genomic Location	Variant	Amino Acid Change	Functional Domain Affected	Gene Expression Altered	Pathogenicity Prediction
*C2orf91*	1	Chr2:41953024	missense	p.(Arg91Ile)	N	NA	Possibly damaging
*CCL16*	1	Chr17:35978161	missense	p.(Cys60Ser)	Y	NA	Probably damaging
*PAG1*	1	Chr8:80984896	synonymous	p.(Pro252=)	Y	NA	Unknown
*VPS13D*	1	Chr1:12283596	missense	p.(Phe1832Val)	N	-	Probably damaging
*VPS4B*	1	Chr18:63400074	missense	p.(Lys255Thr)	Y	NA	Probably damaging
*ZNF750*	1	Chr17:82830337	synonymous	p.(Pro659=)	N	NA	Unknown
*RIMBP3C*	2	Chr22:21546513	missense	p.(Arg1488Ser)	N	NA	Possibly Damaging
*SPATA31A5*	2	Chr9:60919364	missense	p.(Leu970Phe)	N	-	Possibly Damaging
*CCDC27*	3	Chr1:3752496	synonymous	p.(Ile5=)	N	+	Unknown
*LETM2*	3	Chr8:38400906	synonymous	p.(Leu279=)	Y	+	Unknown
*NTRK2*	3	Chr9:84670796	missense	p.(Trp16Cys)	N	NA	Possibly Damaging

**Table:** 

(b)					
Gene	Area	Genomic Location	Variant	Amino Acid Change	Functional Domain Affected
*CSK*	2	Chr15:74798671	frameshift	p.(Glu25fs)	Y
*TSPAN9*	2	Chr12:3283047	frameshift	p.(Leu218fs)	Y

**Table:** 

(c)					
Gene	Area	Genomic Location	Variant	Gene Expression Altered	IMPACT
*MAP3K2*	1	Chr2:127387525	intron	-	Modifier
*RIPK3*	1	Chr14:24332669 or Chr14:24332869	downstream gene	+	Modifier
*RNPS1*	1	Chr16:2266329	intron	-	Modifier
*SNX32*	1	Chr11:65832561	upstream gene	-	Modifier
*AC138649.1*	2	Chr15:22768761	intron	NA	Modifier
*FAM157B*	2	Chr9:138231054	non-coding transcript exon	+	Modifier
*FANCD2P2*	2	Chr3:11871392	non-coding transcript exon	+	Modifier
*LAIR1*	2	Chr19:54358582	intron	NA	Modifier
*NFAM1*	2	Chr22:42432412	upstream gene	+	Modifier
*TET2*	2	Chr4:105241954	intron	NA	Modifier
*TMEM114*	2	Chr16:8569715	3 prime UTR	NA	Modifier
*MOCS2*	3	Chr5:53109455	5 prime UTR	-	Unknown
*PSMB2*	3	Chr1:35641574	5 prime UTR	NA	Modifier
*RUFY1*	3	Chr5:179608552	intron	-	Modifier
*WDR6*	3	Chr3:49005134	upstream gene	NA	Modifier
*Z82190.2*	3	Chr22:31821630	intron	NA	Modifier

**Table 4:** Differentially Expressed Gene Pathway Analysis. These genes were located in copy number aberrant regions defined as copy number more than 3 or lower 1 and also demonstrated differential expression by RNA seq. Different pathways are implicated in the distinct sections.

Location	Chromosome	Start Position	End Position	Raw Copy Number	Genes	Role in Tumorigenesis
Area1	chr17	81509970	81523847	3.151914	*ACTG1*	Anti-apoptosis, motility [64,65]
Area1	chr17	81887843	81891586	3.151914	*ALYREF*	Genomic stability [66]
Area1	chr14	20455190	20457772	4.883921	*APEX1*	Base-excision repair [49]
Area1	chr17	81867720	81871406	3.151914	*ARHGDIA*	Invasiveness, metastasis [67]
Area1	chr12	7080208	7092607	5.842557	*C1R*	Inflammation [68]
Area1	chr17	79778131	79787983	3.109085	*CBX2*	Transcription [69]
Area1	chr17	50183288	50201632	3.060268	*COL1A1*	Metastasis [70]
Area1	chr17	82078332	82098332	3.562293	*FASN*	Metabolism [71]
Area1	chr7	128830376	128859274	3.66148	*FLNC*	Invasiveness [72]
Area1	chr17	82050690	82057470	3.562293	*GPS1*	COP9 signalosome subunit/ubiquitin-proteasome pathway
Area1	chr19	11164266	11197791	7.563794	*KANK2*	Cytoskeleton formation [73]
Area1	chrX	54807598	54816012	3.320925	*MAGED2*	Cell-cycle regulator [74]
Area1	chrX	55452104	55453566	3.320925	*MAGEH1*	Proliferation [75]
Area1	chr7	100092727	100101940	4.16605	*MCM7*	Proliferation [76]
Area1	chr14	22836556	22849027	4.136412	*MMP14*	Invasiveness, metastasis [77]
Area1	chr14	39175182	39183218	3.038443	*PNN*	Splicing [51]
Area1	chr9	107283136	107332194	14.61502	*RAD23B*	Nucleotide-excision repair [53]
Area1	chr18	49488452	49492523	3.095593	*RPL17*	Ribosome biogenesis, protein translation [61]
Area1	chrX	54814369	54814497	3.320925	*SNORA11*	Maturation of ribosomal RNA [62]
Area1	chr7	102194075	102194164	4.159154	*SNORA48*	Maturation of ribosomal RNA
Area1	chr2	5692666	5701385	3.929294	*SOX11*	Transcription
Area1	chr17	76734114	76737374	3.109085	*SRSF2*	Splicing [54]
Area1	chr9	35099775	35103195	3.374564	*STOML2*	Anti-apoptosis [78]
Area1	chr17	61399895	61409466	3.52571	*TBX2*	Transcription [79]
Area1	chr19	58544090	58550722	3.012426	*TRIM28*	Proliferation [80]
Area1	chr9	35056063	35073249	3.374564	*VCP*	Protein degradation [81]
Area1	chr7	101162508	101165593	4.159154	*VGF*	Transcription [82]
Area2	chr2	47335314	47335514	4.114423	*BCYRN1*	Transcription [56]
Area2	chr6	73515749	73523797	3.582945	*EEF1A1*	Translation [57]
Area2	chr19	3976055	3985469	3.359182	*EEF2*	Translation [58]
Area2	chr1	150574550	150579738	4.140715	*MCL1*	Anti-apoptosis [83]
Area2	chr1	151399533	151401944	4.140715	*PSMB4*	Proteasomal function [59]
Area2	chr11	67583594	67586660	3.211531	*GSTP1*	Metabolism [84]
Area2	chr15	65296050	65296166	3.976034	*RNU5A-1*	RNA processing
Area2	chr15	65304676	65304792	3.976034	*RNU5B-1*	RNA processing
Area2	chr7	148983754	148983856	3.383375	*RNY3*	RNA processing
Area2	chr13	27251308	27256691	6.141368	*RPL21*	Ribosome biogenesis, protein translation
Area2	chr9	19375714	19380254	3.739665	*RPS6*	Ribosome biogenesis, protein translation
Area2	chr2	24273613	24273741	4.326829	*SCARNA21*	RNA processing
Area2	chr15	78091171	78091297	3.898802	*SNORA63*	Maturation of ribosomal RNA
Area2	chr1	12221147	12221271	3.552826	*SNORA70*	Maturation of ribosomal RNA
Area2	chr2	10446713	10446849	4.496897	*SNORA80B*	Maturation of ribosomal RNA
Area2	chr12	124911603	124917368	3.034233	*UBC*	Ubiquitin homeostasis [85]
Area3	chr16	28823034	28837237	5.159031	*ATXN2L*	Stress granule regulator [86]
Area3	chr9	136862118	136866286	3.830137	*EDF1*	Transcription
Area3	chr11	2129111	2141238	7.774932	*IGF2*	Proliferation [87]
Area3	chr11	2608327	2699994	7.774932	*KCNQ1OT1*	Transcription [88]
Area3	chr11	2134133	2134209	7.774932	*MIR483*	Transcription [89]
Area3	chr9	127447673	127451405	3.212283	*RPL12*	Ribosome biogenesis, protein translation
Area3	chr19	49487553	49492308	3.051258	*RPL13A*	Ribosome biogenesis, protein translation
Area3	chr19	48615327	48619536	3.174325	*RPL18*	Ribosome biogenesis, protein translation
Area3	chr1	6181268	6209389	3.792397	*RPL22*	Ribosome biogenesis, protein translation
Area3	chr17	74203581	74210655	3.363835	*RPL38*	Ribosome biogenesis, protein translation
Area3	chr11	809646	812880	3.117378	*RPLP2*	Ribosome biogenesis, protein translation
Area3	chr19	49496364	49499689	3.051258	*RPS11*	Ribosome biogenesis, protein translation
Area3	chr19	39433206	39435948	3.408557	*RPS16*	Ribosome biogenesis, protein translation
Area3	chr16	1962051	1964860	3.301972	*RPS2*	Ribosome biogenesis, protein translation
Area3	chr19	8321157	8323340	3.044231	*RPS28*	Ribosome biogenesis, protein translation
Area3	chr17	76557765	76565348	3.374444	*SNHG16*	Transcription [90]
Area3	chr16	1962333	1962466	3.301972	*SNORA10*	Maturation of ribosomal RNA
Area3	chr2	30187433	30187566	3.83836	*SNORA10B*	Maturation of ribosomal RNA
Area3	chr9	136726104	136726234	3.830137	*SNORA17B*	Maturation of ribosomal RNA
Area3	chrY	16138247	16138379	3.968437	*SNORA20*	Maturation of ribosomal RNA
Area3	chr16	1965183	1965310	3.301972	*SNORA78*	Maturation of ribosomal RNA
Area3	chr19	10109756	10109835	5.45924	*SNORD105B*	Ribosomal RNA modification [63]
Area3	chr19	49490614	49490699	3.051258	*SNORD33*	Ribosomal RNA modification
Area3	chr14	21397291	21397401	3.835309	*SNORD8*	Ribosomal RNA modification
Area3	chr14	21392149	21392253	3.835309	*SNORD9*	Ribosomal RNA modification

## Supplementary Materials

The following are available online at https://www.mdpi.com/2073-4425/11/5/499/s1, Supplemental Table S1: Comprehensive Genomic Information for Single Nucleotide Variants.
